# The Sources of Reactive Oxygen Species and Its Possible Role in the Pathogenesis of Parkinson's Disease

**DOI:** 10.1155/2018/9163040

**Published:** 2018-09-02

**Authors:** Minrui Weng, Xiaoji Xie, Chao Liu, Kah-Leong Lim, Cheng-wu Zhang, Lin Li

**Affiliations:** ^1^Key Laboratory of Flexible Electronics (KLOFE) & Institute of Advanced Materials (IAM), Jiangsu National Synergetic Innovation Center for Advanced Materials (SICAM), Nanjing Tech University (Nanjing Tech), 30 South Puzhu Road, Nanjing 211816, China; ^2^Department of Physiology, Yong Loo Lin School of Medicine, National University of Singapore, Singapore 117593

## Abstract

Parkinson's disease (PD) is the second most common neurodegenerative disorder characterized by progressive loss of dopaminergic neurons in the substantia nigra. The precise mechanism underlying pathogenesis of PD is not fully understood, but it has been widely accepted that excessive reactive oxygen species (ROS) are the key mediator of PD pathogenesis. The causative factors of PD such as gene mutation, neuroinflammation, and iron accumulation all could induce ROS generation, and the later would mediate the dopaminergic neuron death by causing oxidation protein, lipids, and other macromolecules in the cells. Obviously, it is of mechanistic and therapeutic significance to understand where ROS are derived and how ROS induce dopaminergic neuron damage. In the present review, we try to summarize and discuss the main source of ROS in PD and the key pathways through which ROS mediate DA neuron death.

## 1. Introduction

Parkinson's disease (PD) is an age-dependent, progressive neurodegenerative disease, characterized by selective loss of dopaminergic (DA) neurons residing in an area of the midbrain known as the substantia nigra [1, 2]. As the second most common neurodegenerative disease, PD remains incurable, which might be underlined by the fact that mechanism for PD pathogenesis is not fully illustrated.

With the intensive studies, it is now widely accepted that genetic background, environment factors, and aging are the key contributors of PD pathogenesis. In recent years, some PD-associated genes have been identified, including *α*-synuclein (SNCA), PTEN-induced putative kinase 1 (PINK1), parkin, DJ-1 (PARK7), and leucine rich repeat kinase 2 (LRRK2), mutations of which lead to the familial forms of PD (early-onset) [3]. Even in the rest of 90% of the sporadic cases of PD, mutations of those genes could also increase the PD susceptibility [4]. Environmental factors such as heavy metals, drugs, and exposure to neurotoxic compounds can induce PD via interfering dopamine transporter activity, dopamine metabolism, mitochondrial function, and proteasome activity [5–7]. Aging could result in misfolding of proteins as well as mitochondrial dysfunction, which are all closely related to the PD pathogenesis [8]. Although the underlying mechanisms of neuronal degeneration in PD remain to be better understood, it is well established that all of the PD-related factors mentioned above can cause excessive generation of ROS [9].

ROS, as the by-products of cellular metabolism, are defined as a group of reactive molecules derived from molecular oxygen, which include superoxide anion (O_2_^−^), hydroxyl radical (·OH), and hydrogen peroxide (H_2_O_2_) [10]. ROS are essential for maintaining many physiological processes such as apoptosis, autophagy, and immunological defense [11]. But if the balance between production and elimination of ROS is disturbed, pathogenic consequences such as neurodegeneration would happen [12].

**Figure 1:**
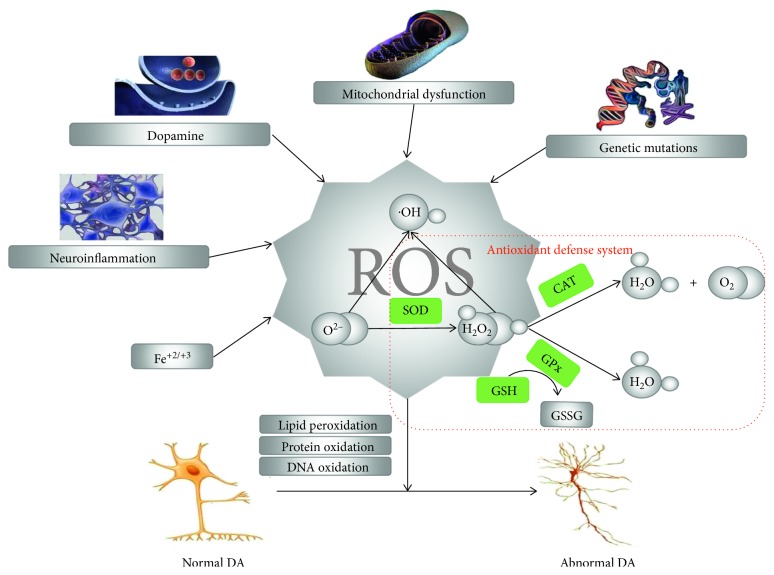
Schematic pathway of ROS generation and induction of DA neurons death. Mitochondria dysfunction, dopamine, neuroinflammation, iron, and genetic mutations solely or synergistically induce ROS generation, which could induce dopaminergic neurons death via protein, lipid, and DNA oxidation.

In this review, we will focus on discussing how the PD-associated factors induce ROS generation and how ROS lead to dopaminergic neuron death in PD (Figure 1).

## 2. ROS and PD-Associated Factors

Numerous evidences suggest that PD-associated factors such as genes mutation, mitochondrial dysfunction, dopamine auto-oxidation, neuroinflammation, iron accumulation, and external toxicants accumulation, all could induce ROS generation.

### 2.1. PD-Related Genetic Mutations and ROS

It has been recognized that the genetic mutations such as *α*-synuclein, PINK1, parkin, DJ-1, and LRRK2 are causative factors of the familial forms of PD [13, 14]. Mutation or multiplication of the *α*-synuclein gene facilitates the accumulation of *α*-synuclein, which is a major component of Lewy bodies, the pathological hallmark of PD [15]. It was indicated that accumulation of *α*-synuclein caused oxidative stress by two parallel pathways: directly stimulating the generation of excessive ROS or indirectly interfering scavenge of damaged mitochondria from which majority of ROS were derived [16, 17]. PINK1 is the kinase that could phosphorylate and activate parkin in the process of damaged mitochondria clearance by autophagy, which exerts neuroprotection against ROS overproduction [18]. It was reported that loss of PINK1 or parkin induced mitochondrial dysfunction and consequent overproduction of ROS, while overexpression of PINK1 or parkin protected against ROS-induced cell death [19, 20]. Parkin is an E3 ubiquitin ligase, and loss of function leads to autosomal recessive PD [21]. Mutation of parkin impairs its function in the elimination of damaged mitochondria, the latter generated ROS [22]. DJ-1 is a small compact protein that localized on the outer mitochondrial membrane (OMM). The sulfhydryl group of DJ-1 could react with ROS to form the cysteine sulfinic acid, which functions as a ROS quencher [23]. Loss of DJ-1 renders increased ROS levels and ultimately caused dopaminergic neuron death [19]. LRRK2 is a large multidomain protein and its mutation leads to autosomal dominant PD. A proposed mechanism for the increased vulnerability of LRRK2 mutant cells to oxidative stress is via the kinase-dependent interaction between LRRK2 and dynamin-like protein (DLP1), which facilitates DLP1 translocation to mitochondria and subsequent mitochondrial fission [24, 25]. Another mechanism is through the interaction of LRRK2 with peroxiredoxin 3 (PRDX3), which is a mitochondrial member of the antioxidant family of thioredoxin peroxidases. Mutations in the LRRK2 kinase domain increase phosphorylation of PRDX3 leading to decreased peroxidase activity, increased ROS production, and increased cell death [26, 27]. Notably, postmortem analysis of brains from PD patients carrying the G2019S mutation in the kinase domain of LRRK2 has shown marked increase in phosphorylated PRDX3 compared to normal brains [28].

### 2.2. Mitochondrial Dysfunction and ROS

Mitochondria are known as the “power houses” of cells, the place generating adenosine triphosphate (ATP) through oxidative phosphorylation (OXPHOS) [29]. During ATP production, ROS also generate from the electron transport chain [30]. The ROS from complex I are released to the mitochondrial matrix, while the ROS from complex III are released to both the mitochondrial matrix and the inner membrane space (IMS) [31]. Mitochondrial dysfunction leads to increased ROS generation; in return, ROS are also harmful to the electron transport chain itself, leading to even higher production of ROS [32, 33]. It was suggested that mitochondria-induced overproduction of ROS was a key factor responsible for cell death and the progression of late-onset neurodegenerative diseases, particularly in idiopathic PD [32, 34]. Mitochondrial dysfunction leads to the deficiency of ATP, which is indispensable especially to dopaminergic neurons to propagate electrical signals, maintain ionic gradients and secrete dopamine [35]. The fact that the activity of the mitochondrial electron transport chain in the substantia nigra of PD patients was decreased compared with age-matched controls, further supported the role of mitochondrial dysfunction in PD [36]. In summary, mitochondrial dysfunction can cause PD though the overproduction of ROS, which underlines the dopaminergic neuron death in PD.

### 2.3. Dopamine and ROS

Dopamine (DA), the neurotransmitter produced from DA neurons, is responsible for the regulation of excitatory and inhibitory synaptic transmission for ensuring smooth coordinated movement [37]. The movement disorder displayed in PD patients is basically underlined by the deficiency of DA. Noteworthy, dopamine is an unstable molecule that may auto-oxidize to form quinones and H_2_O_2_ [38, 39]. H_2_O_2_ could react with iron or oxygen to form more active ˙OH [40]. DA quinones could react with the sulfhydryl groups of the cysteine in proteins, particularly glutathione (GSH), a ROS scavenger, resulting in lower GSH levels, and higher ROS level [41]. In addition, ROS, especially H_2_O_2_, are generated as by product in the process of dopamine oxidative metabolism by monoamine oxidases B [42, 43]. Besides the synthesis and degradation, the transport and storage of dopamine also contribute to elevated ROS production. Dopamine is synthesized in the cytosol and rapidly stored into synaptic vesicles for providing a stable environment for DA before released out [15], which is dependent on vesicular monoamine transporter 2 (VMAT2). Dopamine reuptake, occurred with the help of dopamine active transporter (DAT), is essential for precisely tuning the dopamine level in synaptic cleft [44]. Obviously, any perturbation to the storage and reuptake of dopamine would elevate cytoplasmic dopamine, which enhances the susceptibility to be oxidation. Consist with that, mutant *α*-synuclein, which linked to inherited forms of PD, is associated with enhanced dopamine reuptake and down regulates VMAT2 [45]. In addition, DAT is involved in dopamine neurotoxicity by reuptake dopamine from extracellular space to cytosol leading to accumulation of dopamine [46]. Conclusively, dopamine is an unstable molecule and prone to auto-oxidize in cytoplasm. Any perturbation elevating cytoplasmic dopamine can increase dopamine auto-oxidization and subsequently ROS and eventually PD pathogenesis.

### 2.4. Neuroinflammation and ROS

Neuroinflammation is a protective response of nervous system to various kinds of tissue insults and damage. It would induce release of trophic factors and ROS to protect against stimulus so as to facilitate the regeneration and the repair [47]. Once inflammation is overwhelmed, it would cause accumulation of ROS and consequently cell death [48]. A large body of research shows that chronic inflammation involves in chronic neurodegenerative diseases, particularly the pathogenesis of PD.

Microglial cells, resident immune cells in the central nervous system (CNS), are main participants of the inflammatory response. Activated microglia releases various cytokines and chemokines to initiate corresponding processes to recruit additional microglia and leukocytes to the site of injury [49]. Cytokines such as, TNF-*α*, IL-1*β*, and IFN-*γ*, are proinflammatory, which will activate NADPH oxidases (Nox). Nox2, one isoform of Nox, is mainly expressed in the nervous system involved in the production of ROS as a result of the catalyzing the electron transfer from NADPH to oxygen [50]. In addition, TNF-*α* could cause the depletion of endogenous antioxidants such as GSH of DA neurons, which renders DA neurons more susceptible to ROS [51]. IL-1*β* causes aberrant mitochondrial membrane potential and the depletion of ATP through facilitating the formation of peroxynitrite, ultimately leading to mitochondria dysfunction and consequent increased ROS [52, 53]. Beside cytokines and chemokines, microglia can also be activated by endogenous proteins such as *α*-synuclein [54]. *α*-Synuclein directly promotes activation of Nox_2_ in microglia leading to a burst of ROS. Conclusively, cytokines and chemokines released by microglia can induce NAPDH oxidase activity, which are capable of markedly enhancing the level of ROS and therefore PD pathogenesis.

### 2.5. Iron and ROS

Iron accumulation is another important hallmark of PD, which has been supported by multiple of evidences, especially increased iron level observed in the substantia nigra of PD patients compared to age-matched controls [55]. Iron is indispensable for many fundamental biological processes, but excessive iron is cytotoxic. Neurons therefore tightly regulate iron levels via controlling both iron uptake and iron storage. As established, the homeostasis of cellular iron is coordinated mainly by two iron regulatory proteins (IRP1 and IRP2) [56, 57], which could bind to DNA iron-response elements (IREs) and regulate their translations [58]. With aging, the regulation machinery of iron tends to be compromised and abnormal iron accumulation and increased free iron concentration subsequently occurred [59].

Excessive iron ions can cause an exacerbated ROS production via Fenton and Haber–Weiss reactions. Iron also catalyzes the conversion of excess dopamine to neuromelanin, during which ROS are generated [60]. Consistent with that, N-acetyl-l-cysteine (NAC), an antioxidant, which could decrease iron levels, showed neuroprotective effect in PD models [61]. Moreover, desferrioxamine (DFO) and VAR10303 (VAR), two kinds of iron chelator, reduced the ROS and rescued the MPTP induced PD mouse phenotypes [62, 63]. Collectively, iron can also contribute to pathogenesis of PD via aggravating ROS production.

## 3. Pathological Role of ROS in the PD Pathogenesis

In cells, ROS are strictly regulated by antioxidant defense systems, which mainly consist of superoxide dismutase (SOD), glutathione peroxidase (GPx), catalase (CAT), ascorbic acid (vitamin C), *α*-tocopherol (vitamin E), and GSH [64] (Table 1). Once the formation of ROS overwhelms the antioxidant defense system, oxidative stress will be induced. As motioned above, various PD causative factors can lead to excessive ROS generation, which further emphasizes the pivotal role of ROS in the PD pathogenesis. ROS participated in PD pathogenesis involving the peroxidation of lipid, protein, and nucleic acid [65].

### 3.1. ROS-Induced Lipid Peroxidation

Lipid is the main component of the membrane for cell as well as the organelles, such as mitochondria and nuclear. Lipid, especially polyunsaturated fatty acids, is very vulnerable to the attack of ROS [66]. A hydrogen moiety of unsaturated carbon of polyunsaturated fatty acids could easily be attacked and consequently captured by ROS to form water, leaving an unpaired electron on the polyunsaturated fatty acids, which was converted into a peroxyl radical [67]. Once formed, peroxyl radicals would eventually produce malondialdehyde (MDA), 4-hydroxynonenal (4-HNE), and other toxic products [68, 69]. It was suggested that MDA was the major mutagenic and carcinogenic product of lipid peroxidation, whereas 4-HNE was less mutagenic and carcinogenic but the most toxic [70]. 4-HNE could trigger caspase activation and ultimately cause neuronal apoptosis [71]. In addition, 4-HNE could also reduce the GSH levels via interplaying with sulfhydryl groups [72]. Peroxided lipid reacts with polyunsaturated fatty acids leading to further oxidation, ultimately disrupting plasma membranes [73]. Accordingly, ROS-induced lipid peroxidation can cause neuronal damage and contribute to PD progression.

### 3.2. ROS-Induced Protein Oxidation

It has been demonstrated that ROS initiates protein oxidation by two parallel pathways: directly inducing protein chain and side chain oxidation and indirectly inducing protein oxidation in the process of lipid peroxidation and glycosylation [74, 75]. Protein oxidation includes the cross-linking and fragmentation of protein and carbonyl group formation [76–78]. It is noteworthy that surface-exposed methionine and cysteine residues of proteins are particularly sensitive to oxidation by almost all forms of ROS. ROS-induced protein oxidation potentially effects cell survival via disrupting the active site of enzymes and consequently protein-protein and protein-DNA interactions [79]. It was demonstrated that loss function mutation in DJ-1, one familial PD-related gene, leaded to protein oxidative damage [80]. Supplementation of antioxidant, vitamin C, could decrease the H_2_O_2_ and oxidized protein level [81]. Therefore, protein oxidation by ROS involves in PD pathogenesis.

### 3.3. ROS-Induced DNA Oxidation

It is acknowledged that OH can bind with DNA molecule, leading to oxidation of bases and the deoxyribose backbone [82]. The key product of DNA oxidation is 8-hydroxy-deoxyguanosine (8-OHdG), which results in transcriptional mutagenesis and generation of mutated species of protein that contributed to PD pathogenesis [83, 84]. Notably, mitochondrial DNA (mtDNA) oxidation by ROS would lead to mtDNA abnormality and consequently trigger the expression of aberrant mitochondrial proteins and mitochondrial dysfunction, collectively exacerbating ROS production [85, 86]. It is therefore unsurprising to note that there is a vicious cycle between mtDNA oxidation and increased ROS production, which ultimately leads to neuronal death and PD pathogenesis.

## 4. Anti-ROS with Compounds for the Therapeutics of PD

In light of the above-mentioned evidence on the crucial role of ROS in the pathogenesis of PD, anti-ROS therapy has been an attractive strategy to counteract the oxidative stress-induced neuronal cell death in PD [87]. Classic antioxidants mainly include vitamin C, vitamin E, Coenzyme Q10 (CoQ10), GSH, NAC, and creatine. Vitamin C and vitamin E are members of antioxidant defense systems. Vitamin E could scavenge hydroxyl and peroxyl radicals, thus protecting against lipid peroxidation [88]. Vitamin C could not only directly remove O_2_^−^ and ˙OH, but also indirectly facilitate vitamin E to counteract overproduced ROS to show neuroprotection in PD [89, 90]. It was reported that a combination of vitamin C and vitamin E administered to patients with early PD may slow the progression of the disease [91, 92]. CoQ10, a constituent of the mitochondrial electron transport chain (ETC), prevented electrons leaking along the ETC which would generate ROS [93]. It was reported that oral administration of CoQ10 in PD animal models and PD patients attenuated mitochondrial dysfunction and deficit of dopamine [94]. Mechanically, CoQ10 acted as antioxidant to scavenge H_2_O_2_ or as a cofactor and activator of mitochondrial uncoupling proteins to decrease the generation of ROS [93, 95]. GSH, the major endogenous antioxidant molecule, was found to reduce in the substantia nigra of PD patients [96]. However, direct administration of GSH did not achieve expected effect of scavenging ROS due to its susceptibility to oxidation by various ROS [97]. NAC, a precursor of GSH, was alternatively utilized to restore GSH levels by providing the rate-limiting substrate for GSH synthesis [98]. Moreover, NAC could also directly act as a scavenger of ROS and ameliorate dopaminergic neuronal loss in PD models [99, 100]. Creatine is a nitrogenous guanidine molecule with antioxidant properties, which could retain mitochondrial dysfunction and protect DA neuron death in PD models [101, 102]. As known, most of the ROS are produced during ATP production though OXPHOS. Resveratrol, a natural polyphenolic compound, is showed to protect against Parkin deficiency-induced mitochondria dysfunction and oxidative stress via activating AMPK/SIRT1/PGC-1*α* axis [103]. Pinocembrin (PB) could mitigate MPP (+) induced SH-SY5Y cells oxidative stress and apoptosis [104].

Nuclear factor erythroid 2-related factor 2 (Nrf2) controls the antioxidant and detoxifying response in mammalian [105]. Recently, it was reported that carnosic acid (CA) exerts antioxidant effects through activation of Nrf2, the latter upregulating expression of some of endogenous antioxidants such as GPx, glutathione reductase (GR) [106]. Moreover, isothiocyanate sulforaphane (SFN), another Nrf2 activator, also displays neuroprotective effects in PD models [107]. All those studies suggest that Nrf2 is a pivotal mediator of cellular antioxidative stress system.

Noteworthy, antioxidants show the promising effect for antagonizing oxidative stress in animal PD models, and they do not display the equivalent efficacy in clinical trials. More work need to do before antioxidant could be applied for PD treatment in clinic.

## 5. Conclusions

PD is the second most common neurodegenerative disorder, and the mechanisms of neuronal degeneration in PD are poorly known and remain to be fully illustrated. It is widely accepted that genetic mutations, mitochondrial dysfunction, dopamine auto-oxidation, neuroinflammation, and iron accumulation contribute significantly to the pathogenesis of PD. Interestingly, all of the PD-related factors can cause excessive generation of ROS. Once ROS overwhelm antioxidant defense systems, excess ROS can induce lipid peroxidation, protein oxidation, and DNA oxidation to trigger PD-related cell loss in the SN. In the future, the molecular signal pathway of ROS inducing PD pathogenesis needs be further explored. Antioxidants which could be utilized for PD treatment should be developed.

## Figures and Tables

**Table 1:** Antioxidant defense systems and proposed mechanisms against ROS.

Classifications	Antioxidants	Functions
Enzymatic antioxidant defenses	Superoxide dismutase (SOD)	SOD catalyzes two O^2−^ anions to convert into a molecule of H_2_O_2_ and oxygen 2 O^2−^ + 2H^+^ → H_2_O_2_ + O_2_
	Glutathione peroxidase (GPx)	GPx, a family of multiple isoenzymes containing selenium, catalyzes the degradation of H_2_O_2_ and lipid peroxides. Moreover, GPx can utilize GSH as an electron donor for the reduction of peroxides [64].
	Catalase (GPx)	Catalase, mainly existing in peroxisomes, is responsible for converting H_2_O_2_ into water 2 H_2_O_2_ → 2 H_2_O + O_2_

Nonenzymatic antioxidants	Ascorbic acid (vitamin C)	Vitamin C, a water-soluble antioxidant, is capable of removing ROS by electron transfer. In addition, vitamin C can act as a cofactor for antioxidant enzymes [88]; [90]
	*α*-Tocopherol (vitamin E)	Vitamin E, a lipid-soluble antioxidant, can attenuate the effects of peroxide. In particular, it can protect against lipid peroxidation in cell membranes [88]
	Glutathione (GSH)	GSH, in its reduced form, is known to react with ROS for the removal of ROS. Moreover, GSH is the electron donor for the reduction of peroxides in the GPx reaction [64]
